# Rapid Realist Review of School-Based Physical Activity Interventions in 7- to 11-Year-Old Children

**DOI:** 10.3390/children8010052

**Published:** 2021-01-16

**Authors:** Emmanuel Defever, Michelle Jones

**Affiliations:** 1 Health and Social Sciences, Faculty of Sport, Southampton Solent University, Southampton SO14 0YN, UK; e.t.defever@gmail.com; 2 Resilience and Human Performance Research and Knowledge Exchange Group, Plymouth Marjon University, Plymouth PL6 8BH, UK

**Keywords:** physical activity, school setting, socioecological model, intervention, realist synthesis

## Abstract

Meta-analysis of physical activity interventions in school settings have revealed low efficacy and that there is a need to explore implementation fidelity. The aim of this rapid realist review was to determine, what physical activity interventions in school settings for children aged 7- to 11-years-old works, for whom, and in what circumstances. The realist synthesis was conducted following RAMESES guidelines. Relevant studies were identified following a systematic search process and data from 28 studies was extracted for evidence to form context-mechanism-outcome configurations that were clustered and refined. Using the five-level socioecological model, the program theories were classified into the levels of intrapersonal (child), interpersonal (teachers), institutional (program content, school administration, and school environment), community (home and neighborhood), and policy. The school level led to most context-mechanism-outcome configurations related to school leadership and policy, workforce structure, program characteristics, and school environment. At each level, we identified features of interventions, alongside implementation considerations that might work to promote efficacy and sustainability. The need to recognize the school environment as part of a complex system with multi-level interaction and influences was a key finding. In line with realist philosophy, the researchers encouraged primary research to confirm, refute, and refine the program theories presented.

## 1. Introduction

Physical activity is associated with physiological, developmental, mental, cognitive, and social health benefits in young people [1,2,3]. Consequently, recently updated global physical activity guidelines recommend children aged 5 to 17 years engage in an average of 60 min a day of moderate-to-vigorous physical activity [4]. It has been estimated that approximately 80% of young people worldwide do not meet these physical activity guidelines [5]. Recent evidence suggests a decline in physical activity from early childhood [6,7,8]. For example, a cross-sectional study incorporating 10 countries identified an annual decrease of 4.2% moderate-to-vigorous physical activity after 5 years of age [7]. It is therefore imperative to promote physical activity and intervene early in childhood prior to the decline in physical activity. Children compulsorily spend 40% of their waking time at school [9] and the school setting provides a location for interface with all children and so is an ideal environment for population-based physical activity interventions [10].

Two recently published systematic reviews and meta-analysis of school-based interventions identified no significant effect size for daily moderate-to-vigorous physical activity across 17 randomized controlled trials in the 6- to 18-year-old age range [11] or a significant moderate effect for daily moderate-to-vigorous physical activity but with low precision and considerable inconsistency across 10 experimental studies in 5- to 11-year-old children [12]. Current intervention approaches seem relatively ineffective at promoting whole day physical activity and thus it is timely to explore implementation fidelity [11,13]. Published systematic reviews provide limited insight into the complex causal pathways that may underpin intervention effectiveness [14] or mechanisms that explain how interventions and participants interact to produce observed outcomes [15]. Schools are complex social structures with unique qualities that can influence intervention fidelity and do not always allow for programs to be transferred from one school to another with predictable results [16]. A realist review explores causal mechanisms of intervention effects under different contextual circumstances and might provide new insights in terms of school-based physical activity interventions. For example, Brown et al. [14] conducted a dual meta-analysis and realist synthesis to understand the effectiveness of family-based physical activity interventions and a realist synthesis revealed novel insights including that interventions focusing on something other than the health benefits of physical activity or weight loss, such as improving quality family time, appeared to be an effective mechanism for changing physical activity behavior.

Realist methodology is a theory-driven approach that generates a set of program theories in context-mechanism-outcome (CMO) configurations to provide a scientific basis for the transferability of the theories developed and tested [17,18]. The realist methodology provides a means to deal with intervention heterogeneity and make inferences about context and effectiveness [19,20]. Realist reviews can complement what is already known from systematic reviews and supplement the knowledge by exploring underlying causalities [21]. There is relatively little information on the underlying mechanisms of change within school-based physical activity interventions; indeed, few studies have identified a theoretical framework to underpin intervention design. Existing intervention studies that describe a theoretical underpinning have typically identified social cognitive theories [22,23,24,25,26], whereas Beets et al. [27] suggested that interventions underpinned by behavioral theories are largely misplaced in assuming children are autonomous agents in their physical activity decision-making. A small number of studies reported designing interventions based on a social-ecological model that integrates intra- and inter-personal influences alongside the impact of the organization, community, and wider public policy [28,29,30,31,32,33]. Furthermore, there are some national frameworks for physical activity promotion within school settings that are typically multi-component and recognize levels of influence similar to the socioecological model, e.g., the Comprehensive School Physical Activity Program in the United States of America. The socioecological model was developed as an ecological model for health promotion, focusing on both individual and social factors and recognizing the dynamic interrelations among individual and environmental factors. As such, the socioecological model and realist methodology share similarities in that they recognize that behavioral change interventions can be messy and complex [34], with a multitude of interactions between stakeholders and the target population, all of whom share a certain context governed by policies.

A rapid realist review has the potential to inform the development of a conceptual framework by exploring intervention characteristics that generate observed changes (i.e., mechanisms). When the mechanisms are triggered under certain circumstances (i.e., context), they ultimately change the decision-making or behaviors of the target population (i.e., outcomes, intended or unintended) [35]. Exploring complex mechanisms that are often hidden can help researchers understand and explain observed outcomes (intended and unintended) within a phenomenon [36]. The rapid realist review can be seen a complementary analysis to systematic review and meta-analysis to explore the contexts that trigger certain mechanisms and the resultant outcomes that can be hidden within systematic reviews. The aim of this rapid realist review was to determine, within a socioecological model, what works, for whom, and in what circumstances for physical activity interventions in school settings for children aged 7- to 11-years.

## 2. Materials and Methods

This rapid realist review is a complimentary analysis accompanying a published systematic review and meta-analysis that details the full methodology [12]. In brief, the review was registered with the prospective register of systematic reviews (PROSPERO, CRD42017082184). A literature search was conducted to identify peer-reviewed intervention studies of any methodological design that promoted physical activity in school settings in children aged 5 to 11 years. A structured electronic bibliographic search of five databases (ERIC, MEDLINE, PsychINFO, SPORTDiscus, and Web of Science) was used to retrieve articles published in the English language through 30 June 2017. The search strategy combined multiple keyword search terms agreed to a priori. The search terms focused on 4 key elements: (1) outcome measure; (2) study population; (3) study type; and (4) setting. A two-step screening process was used to determine whether each study met the inclusion criteria. Studies were included if they: (1) involved children of primary/elementary/middle school age, e.g., 5- to 11-years-old; (2) reported on an intervention that lasted at least 4 weeks, was implemented within a school environment, and was targeted at physical activity; and (3) reported an objectively assessed measure of physical activity. Following title and abstract screening of 1115 records, the removal of duplicates (*n* = 584) and articles that did not meet the inclusion criteria (*n* = 419) resulted in 112 studies remaining. Two independent reviewers assessed the full text of the remaining 112 studies against the inclusion criteria, resulting in a further 52 studies’ being excluded. The systematic review, therefore, included 57 original studies. The quality of the included studies was assessed by two independent reviewers using the mixed methods appraisal tool [37], which has content-validity for each domain. Items were developed from the literature, as were consultations and workshops with experts [37,38,39].

This rapid realist review was conducted using the RAMESES quality and publication guidelines for realist synthesis [21]. The literature was synthesized to derive the hypothesized context and mechanisms necessary to elicit a specified outcome. Data extraction (e.g., verbatim sections of text) for the review included “gleaning stages” conducted initially by one reviewer (ED) and then confirmed/challenged by a second (MJ) until consensus was reached. Each of the full-text studies was reviewed for any evidence, description, or insight contributing to forming either full or partial CMO configurations and focused specifically on those articles that included children in the 7- to 11-year-old age range as participants. A total of 28 full-text articles provided sufficient information to describe outcome patterns and thus contributed to the realist rapid review. The relevant information was extracted verbatim and grouped into clusters of similar topics. Where appropriate, theories cited in the included interventions were noted to provide additional insight into possible or missing mechanisms. The second process included arranging the extracted verbatim into CMO configured statements. Within each cluster, CMO configurations were then distilled for conciseness and simplicity. The third gleaning involved mapping the CMO configurations for visual clarity within the levels of the socioecological model. Finally, the fourth process involved refining the CMO configurations to concisely reflect the evidence from included studies.

## 3. Results

### 3.1. Description of Studies

The study designs of the 28 studies included randomized controlled trials (RCT, *n* = 12, 43%) [22,40,41,42,43,44,45,46,47,48,49,50], cluster RCTs (*n* = 5, 18%) [23,24,51,52,53], non-randomized control studies (*n* = 8, 29%) [25,29,54,55,56,57,58,59], and descriptive studies (*n* = 3, 11%) [60,61,62]. Quality scores using the mixed methods appraisal tools ranged from 0% (*n* = 5, 18%) [23,45,46,49,50], 25% (*n* = 2, 7%) [41,59], 50% (*n* = 7, 25%) [40,42,44,47,48,54,56], 75% (*n* = 8, 29%) [24,25,29,43,55,57,58,61], and 100% (*n* = 6, 21%) [22,51,52,53,60,62]. Over half of the studies were published from 2011 onwards (*n* = 15, 54%) [22,23,24,29,40,44,45,46,52,54,55,57,59,60,61], whereas 12 studies (43%) were published between 2001 and 2010 [25,42,43,47,48,49,50,51,53,56,58,62] and one study was published before 2000 [41]. The interventions were conducted in the United States of America (*n* = 12, 43%) [24,41,43,44,45,47,48,51,54,55,59,61], United Kingdom, Ireland (*n* = 8, 29%) [22,25,40,49,50,56,58,60], other European zone countries (e.g., Belgium, Denmark, Iceland, the Netherlands, and Switzerland, *n* = 5, 18%) [23,42,46,53,57], and Australia and New Zealand (*n* = 3, 11%) [29,52,62].

Intervention duration ranged from four weeks to two years; 11 studies lasted for approximately one school term (up to 12 weeks or 3 months, *n* = 13, 46%) [22,24,29,40,42,44,47,48,49,55,58,60,62], seven studies (25%) lasted between one term and one academic year [25,46,52,53,54,59,61], and six studies (21%) lasted more than one academic year [23,41,43,45,51,57]. Two were longitudinal follow-up studies [50,56]. Interventions included a variety of approaches such as expanding, enhancing, or extending play time (*n* = 9, 32%) [29,42,44,45,46,49,50,52,58], whole-school approaches (*n* = 8, 29%) [23,25,41,53,54,55,56,59], classroom-based physical activity (*n* = 4, 14%) [22,40,48,51], use of pedometer as an intervention tool (*n* = 3, 11%) [47,60,62], physical education (*n* = 1, 4%) [57], after-school activity (*n* = 1, 4%) [43], active travel to and from school (*n* = 1, 4%) [24], and school policy change (*n* = 1, 4%) [61]. No detail of a theoretical framework was described for the intervention in a high proportion of studies (*n* = 17, 61%) [40,41,42,44,46,47,48,49,50,51,52,54,57,58,60,61,62]. Five studies (18%) reported the application of social cognitive theory [23,24,25,43,56], three studies (11%) reported intervention design around the socioecological model [29,45,53], one study (3%) reported use of a behavioral change model [22], one (3%) reported theories of organizational change and innovation [55], and one reported an unspecified behavioral theory [59].

### 3.2. Literature Derived Program Theories

The studies demonstrated variation in the context within which interventions were conducted, the intervention strategies employed, and the mechanism targeted. Despite the heterogeneity of the intervention types, the studies contributed to generating an extensive list of initial program theories. The CMO configurations from each study were distilled to draw out patterns to explore insights into the architecture of school-based physical activity interventions.

Using the five-level socioecological model by McLeroy et al. [63], the list of initial program theories were classified into the levels of intrapersonal (i.e., child), interpersonal (i.e., teachers), institutional (i.e. program content, school administration, school environment), community (i.e., home and neighborhood), and policy. At an individual level (child), Table 1 summarizes the key CMO configurations that are primarily related to the use of pedometers, goal setting, and rewards. The context of how pedometers were implemented triggered different mechanisms and outcomes. Table 2 summarizes the key CMO configurations at an inter-personal level where the key context was the classroom teacher, training, resources, and involvement required by the teacher. Adequate training and resources triggered mechanisms that supported implementation fidelity of interventions. The institutional or school level led to the greatest number of CMO configurations. There were three sub-levels: program characteristics, school leadership and administration, and school environment. Table 3 summarizes the CMO configurations. In summary, the contexts that led to higher implementation fidelity and efficacy included school leadership support, limited impact on stretched resources, a workforce structure with dedicated physical activity staff, maximizing utilization of the school playground with trained workforce, and approaches to non-controllable factors such as adverse weather. In comparison, ineffective or inconsistent implementation and program outcomes typically occurred when time and resources were stretched and there was no school leader support, especially when training for staff is not provided and workforce structures do not prioritize physical activity. There were relatively few CMO configurations at the community and policy level, as summarized in Table 4. This reinforces the importance of parental support and the challenges of educational policy that prioritizes academic achievement.

## 4. Discussion

### 4.1. Intrapersonal Level: Child

At an individual level, the main strategy implemented was goal setting that was typically at a class-level as opposed to individual target setting [25,54,62]. This type of goal setting seemed to shift the sense of success or failure from individuals to the whole class, as a team, which in turn created a positive social and peer supportive environment that led to increased physical activity [25,54,56]. In relation to the question of what works and for whom, goal setting seemed to lead to different outcomes dependent upon group characteristics. One study reported a differential response to a virtual walk challenge, finding that only those with lower initial physical activity significantly increased physical activity, especially girls [62]. Another study reported that a goal setting daily step challenge led to a differential response based on age with grade 3 children (8 to 9 years) experiencing a significant reduction physical activity in contrast to grade 6 children (11 to 12 years) who significantly increased physical activity [54]. It was speculated that children in the younger age range do not have sufficient autonomy to make their own life choices independently, as parents have the overall responsibilities in shaping their behavior. A child participant in Gorely et al. [56] (p. 9) commented that “most of the time (she was) not allowed to run around and do things because (her) mum wants to keep (her) safe.” These interactions influence child behavior and are reflected in the community level CMO configurations. Younger children may be hampered or encouraged to apply their newly acquired behavioral change skills outside the school environment dependent upon parental influence [64,65]. This differential response highlights the weakness of methods of statistical analysis that aggregate findings. The headline program theory suggests goal setting at the class level may be an effective aspect of physical activity programs in older children (10–11 years) and especially in those with low initial fitness.

### 4.2. Interpersonal Level: Teacher

The main stakeholder at the interpersonal level of the socioecological model was the class teacher and the overarching mechanisms related to the teachers perceived autonomy and empowerment to facilitate physical activity. Class teachers are crucially positioned in the school-based intervention as “agents of change” [66]. Presenting teachers with interventions that fit with their schedules, curriculum, and beliefs/values about teaching is important for successful implementation [67]. When implemented in a way that triggers positive response mechanisms, class teachers delivered physical activity interventions that led to increased physical activity, including stand-alone class physical activity breaks [40,48,61], physically active learning [22], or class physical activity breaks as part of a multi-component program [23,25,59]. McMullen et al. [68] identified three main factors that contributed to teachers adopting a physical activity program; 1) the need for classroom control, 2) a preference for physical activity integration with connections to academic content, and 3) the importance of implementation ease and student enjoyment.

The CMO configurations suggested several critical implementation factors, i.e., the initial training and whether this adequately covered the concept, purpose, and requirements of program delivery, as well as the level of autonomy the teacher was permitted by the program to fit around their normal teaching routine. The ability of the teacher to tailor and adapt the program contributes to teacher compliance and their positive receptiveness towards physical activity promotion [22,25,40,69]. If teachers are empowered and autonomous then they are likely to implement the program with high fidelity; this in turn creates a cyclic positive chain reaction whereby children enjoy physically active lessons. Some studies reported additional benefits such as increased time on tasks subsequent to the class’ physical activity breaks [48]. In contrast, if teachers were not provided with adequate training or resources, then the intervention was perceived as a stand-alone and teachers were disempowered to implement the program due to little sense of ownership; this resulted in low fidelity or no uptake of the program. In the current climate of educational policy where academic achievement is typically prioritized, any delivery of interventions with no directly perceived impact on academic targets must conform within the frameworks that demand performance outcomes [70]. Teachers can feel overwhelmed with the amount of teaching that has to be covered in a single day, let alone having the capacity to even consider implementing physical activity programs on top of this learning [61].

### 4.3. Institutional Level: School

The institutional level program theories were divided into three sub-levels: program characteristics, school leadership and administration, and school environment. The head teacher primarily constructs a school culture, but the requirement for the school to achieve high academic standards has a substantial influence on school culture. The emphasis on academic achievement creates barriers to teachers taking on responsibility for anything other than time devoted to core subjects [69]. As such, physical activity programs need to include designs that are low cost, cause minimal disturbance, and are adaptable to the existing school operational routine to have higher implementation fidelity [40,71]. Teachers can perceive delivery of health promotion programs as an additional responsibility and that they are likely to implement in a permissive culture. An interview of a head teacher in the study by Langille and Rodgers [72] (p. 887) commented that “it is just a matter of changing the culture to make sure that everybody thinks that (it is) an important thing to do and not interfering with their language arts lesson or their math lesson and so on and so forth.” The school culture also impacts on the workforce structure; for example, employment of a physical activity specialist can result in children’s perceived support and relevance of physical activity, resulting in potentially effective program implementation [54].

In terms of program characteristics, resource availability was an important context. The school setting has a finite availability of resources and when physical activity programs compete with already stretched school resources the program has low implementation fidelity [47,56]. However, if a physical activity program is set-up with low running costs and minimal resources required, then there is less disturbance and implementation of the program is feasible [40]. The characteristics of the program was another contextual factor grouped into whether the delivery was compulsory (e.g., classroom-based physical activity where participation is mandatory), structured (e.g., adult facilitated playtime, afterschool activities), or unstructured (child facilitated). Compulsory physical activity requires the whole class to participate and thereby was most likely to increase the physical activity of every child with high adherence [22,53]. Similarly, children willingly participate in structured physical activity as the responsibility of initiating, organizing, and resolving conflict during the activity is shifted to the facilitator and children can become passive recipients of the intervention [43]. The unintended outcome of compulsory and structured delivery is that when the organized activity is no longer available, the continuation of physical activity participation is jeopardized [53,56]. Alternately, unstructured physical activity opportunities encourage children to initiate, organize, and engage the activity leading to small but significant increases in physical activity [29,52] and these responses can be supported by appropriate social prompting [44]. Children’s creativity and physical/social development is stimulated by unstructured physical activity, which thus encourages self-directed physical activity [29,52].

The environmental component at the institutional level of the socioecological model identifies ways in which schools can adapt the relatively un-modifiable physical environment to influence children’s physical activity. For example, studies have altered the playground space either by staggering playtimes or installing markings and structures [46,49,50,58]. Staggering playtimes allows more space per child [50] and when the space is allocated for specific activities (e.g., designated zones for football and free play) it allows more space for other activities [46,49]. This type of space zoning disrupts the age-based hierarchy of space occupancy and also reduces gender differences in physical activity during play time [46]. The installation of playground markings or structures seems to require sustained stimuli to maintain children’s motivation to stay active [49,50]. There was an interaction of the socioecological model between the institutional environment and interpersonal levels. Playtime supervisors or teachers on playtime duty impacted the outcomes of interventions. Training playtime supervisors by providing resources such as moveable equipment, game cards, and/or a monthly play theme seemed to act as a stimulus for increased physical activity [45,46,50]. In contrast, there was an unexpected response mechanism by teachers/supervisors who perceived playtime as a social time and chance to catch up with colleagues and thereby focused on the safety aspect only. This, naturally, led to lower implementation fidelity [45]. A final context identified was adverse weather including cold weather, lower daylight hours, and rain. However, support for classroom-based physical activity programs led to a response of maintenance of physical activity in the winter [22].

### 4.4. Community and Policy Level

Previous research has suggested that social support from parents does not mediate child physical activity behaviors [73,74]. Few studies have incorporated outreach components into school-based interventions perhaps due to reported difficulties engaging parents in physical activity programs [75]. Those studies including a home-based element have included activity-based homework [25,56] and active travel [24]. Two studies did identify mechanisms in relation to parental support; one study that trebled PE curriculum time discovered an unintended parental response that out of school activity was not necessary [57] and another study identified parental self-efficacy to enable children to participate and cost-benefit analysis resulted in lower fidelity [24]. To improve the effects of school-based intervention, collaboration with its wider community and families is required [76]. A teacher participant in Gorely et al. [56] (p. 8) commented, “I think a lot of it is home life; if the parents do not push them towards sporting activities then you are fighting a battle straight away in school.” This reinforces that school is only one setting within a broader ecological system in which children operate [33,77,78]. In particular, young children are heavily influenced by parental behavior, an influence [41] connected to perceived neighborhood safety [79,80] and parent’s self-efficacy in relation to physical activity [24,57]. A multifaceted school-based approach involving different stakeholders generally yields more positive outcomes compared with those that target single components such as school, family, or community [81,82,83,84].

There were no studies that intentionally set to adjust the policy level outside of school policy/culture of the socioecological model, although the impact of the educational policy on intervention design and implementation was frequently mentioned at an interpersonal and institutional level. The complexity of policy development, the length of time required to detect perceived impact, and the level of direct influence is not as simple as measuring behavioral change at an individual level [85]. Langille and Rodgers [72] also identified that relatively little research has explored how public policy, community influences, and organizational factors all simultaneously contribute to healthy behaviors among children. Their study identified that school administrators perceived a need for overall direction from the policy level but the responsibility on exactly how to implement physical activity strategies should be determined by the schools. For example, a participating head teacher commented that “the more importance that the government places on a concept the more it will happen” (p. 886). Another noted that “in terms of having that policy, a requirement puts pressure on schools to make sure that we implement it” (p. 886). Policy makers need to recognize that although schools are a means of targeting the vast majority of children, relatively simple interventions at interpersonal or intrapersonal level of the ecological system are unlikely to be effective, as they require some degree of “top down” influence to create a chain reaction across multiple levels [86].

## 5. Conclusions

To date, this is the first realist review of the evidence on school-based physical activity interventions, specifically focusing on children. The synthesis of the evidence highlighted the complex interactions of context, mechanisms, and outcomes at each level of the socioecological model to provide new insights into what works in school-based physical activity intervention, for whom, under what circumstances, how, and why. The findings have identified intervention features and their implementation that might work in a given context, as well as implementation considerations to promote sustainability. A key message from the evidence synthesis is the recognition of the school environment as part of the bigger societal environment experienced by children and other stakeholders. The findings align with the active school framework proposed by Daley-Smith et al. [13], which identified key stakeholders as school leaders, teachers/other staff, children, parents, and wider stakeholders, and the need to create system change. Further, the conceptual framework could be applied within the active school framework, especially in relation to policy and vision, social and physical environment, and seven opportunities for physical activity that could support decisions to promote sustainable physical activity behavior change for all children within the school context. The validity of a rapid review is questioned by some [87,88]; however, there remains a need to achieve a balance between comprehensiveness and timeliness for completing a meaningful evidence synthesis [89,90]. The synthesis process generated a large number of initial program theories that demonstrate the complex and multi-level interactions of influences for school-based interventions. Importantly, future research should consider this complexity in relation to intervention design, implementation consideration, evaluation, and analysis plans. The results of this review contribute to knowledge about the multifaceted interactions that influence how physical activity can be enhanced within a school setting given certain contexts. In line with realist philosophy and approaches, the authors encourage future primary research to confirm, refute, and refine the program theories presented [91].

## Figures and Tables

**Table 1:** Individual level (child) of a conceptual framework for school-based physical activity interventions in children aged 7–11 years of program theories (context-mechanism-outcome).

Context	Mechanism	Outcome	Supporting Evidence
If pedometers are used to set whole class targets (goal setting) and a celebration event is provided.	Then pedometers encourage inter-class competition and create social support.	Positive influence on individual behavior and leads to increased daily step count.	Gorely et al. [25] Oliver et al. [62]
If pedometers are used as a self-monitoring device (individual goal setting).	Then pedometers provide a physical activity currency, which is popular especially for those children with low initial physical activity as it motivates children.	Raised awareness of physical activity and increased step count.	Duncan et al. [60] Gorely et al. [25]
		But, there is a ceiling effect for children with high baseline physical activity.	Kang and Brinthaupt [47]
If pedometers are used without a rewarding system.	Then younger children resent the burden and do not understand goal setting.	Ineffective individual behavior change and no increase in physical activity.	Burns et al. [54]

**Table 2:** Interpersonal level (class teacher) of a conceptual framework for School-Based Physical Activity Interventions in Children Aged 7–11 Years of program theories (context-mechanism-outcome).

Context	Mechanism	Outcome	Supporting Evidence
If teacher training workshops are provided.	Then teachers become empowered and increase delivery confidence and competence. Teachers feel a sense of autonomy and ownership over the program.	Teachers model positive physical activity behavior and there is high program implementation fidelity and increased child physical activity.	Magnusson et al. [23] Donnelly et al. [51]
If physical activity resources and equipment are provided without training.	Then teachers lack autonomy and perceive physical activity program delivery as a stand-alone and additional.	Teachers do not use, or misuse the resources provided.	Huberty et al. [45] Martin and Murtagh [22] Weaver et al. [59]
If the intervention requires substantial teacher involvement,	Then there will be a differential response depending upon willingness and training provided.	Ineffective and/or inconsistent implementation of the physical activity program.	Drummy et al. [40] Huberty et al. [45] Martin and Murtagh [22] Weaver et al. [59]

**Table 3:** Institutional level (school) of a conceptual framework for School-Based Physical Activity Interventions in Children Aged 7–11 Years of program theories (context-mechanism-outcome).

Context	Mechanism	Outcome	Supporting Evidence
If the school leadership implement changes to school level policy to support physical activity.	Then teachers see the high-level support which increases importance of physical activity and enables a whole school approach.	Effective implementation and increased child physical activity.	Gorely et al. [25]
	But, when there are competing academic demands and reduction in high level support.	The Physical activity program loses support and implementation fidelity is low.	Holt et al. [61]
School based resources are stretched, and time is limited. If the physical activity program characteristics require school resources.	Then a low cost and set up that is easy to deliver within the stretched resource base.	Higher implementation of the program and increased child physical activity.	Drummy et al. [40]
	But, if higher costs/time is required the program is viewed as impractical and no time to implement.	Ineffective and/or inconsistent implementation of the physical activity program.	Kang and Brinthaupt [47] Gorely et al. [56] Holt et al. [61]
If the school workforce structure includes a dedicated staff position for physical activity.	Then it increases the chance of program sustainability and continuity	Program implementation, delivery and maintenance are improved.	Burns et al. [54]
If the characteristics include structure and adult supervision of physical activity (e.g., active learning, formal playtime program).	Then the whole class/group engages with the program. But the program will stop when the formal intervention stops.	Children are happy to participate and certain groups, especially girls, increase their physical activity during the intervention. But this is not maintained beyond the formal intervention.	Dzewaltowski et al. [43] Ridgers et al. [49] and [50] Efrat [44]
But if the characteristics of the program is unstructured physical activity (e.g., increased recess time or free play equipment).	Then it stimulates creativity and child autonomy increases with more self-directed physical activity.	More sustained increases in child physical activity and PA maintains challenge.	Hyndman et al. [29] Engelen et al. [52]
If the school playground environment is maximized including staggered lunch times and sectioning areas for specific activities.	Then girls are more likely to occupy play spaces they normally do not and there is an increased choice of physical activity.	Reduced gender differences in physical activity and overall increased child physical activity.	Janssen et al. [46] Ridgers et al. [49,50]
But if the school playground is supervised but otherwise not managed.	Then certain spaces remain occupied by specific groups and there are dominant play characteristics (e.g., football).	Differential intervention outcomes (e.g., of increased play time) by age and gender, older children may reduce physical activity.	Janssen et al. [46] Ridgers et al. [49]
And if training is provided for playground supervisors.	Then supervisors initiate activities and increase use of play equipment.	Sustained stimulus for physical activity and increased child physical activity.	Gorely et al. [25]
But if training is not provided for playground supervisors during an intervention.	The intervention can be seen as interference with a chance to socialize among staff	Intervention program loses support and low implementation fidelity.	Huberty et al. [45]
If the school has approaches related to adverse non controllable factors such as adverse weather.	Then alternative indoor classroom physical activity can take place and disruption is minimized.	Physical activity is sustained and unaffected by adverse weather.	Martin and Murtagh [22]

**Table 4:** Community and policy level of a conceptual framework for School-Based Physical Activity Interventions in Children Aged 7–11 Years of program theories (context-mechanism-outcome).

Context	Mechanism	Outcome	Supporting Evidence
If parental support is not established for physical activity intervention.	Then parents consider a cost-benefit analysis and many have low self-efficacy to engage with the program.	Ineffective implementation and or inconsistent outcomes on child physical activity.	Mendoza et al. [24] Gorely et al. [56] Oliver et al. [62]
If education policy prioritizes academic attainment.	Then teachers focus on academic achievement and if the program does not support academic attainment.	Ineffective implementation of physical activity.	Kang and Brinthaupt [47] Gorely et al. [56] Holt et al. [61]

## Data Availability

Data sharing not applicable.
